# The Prospect of Hydrolytic Enzymes from *Bacillus* Species in the Biological Control of Pests and Diseases in Forest and Fruit Tree Production

**DOI:** 10.3390/ijms242316889

**Published:** 2023-11-29

**Authors:** Henry B. Ajuna, Hyo-In Lim, Jae-Hyun Moon, Sang-Jae Won, Vantha Choub, Su-In Choi, Ju-Yeol Yun, Young Sang Ahn

**Affiliations:** 1 Department of Forest Resources, College of Agriculture and Life Sciences, Chonnam National University, Gwangju 61186, Republic of Korea; ajunahenry@jnu.ac.kr (H.B.A.); mjh132577@naver.com (J.-H.M.); lazyno@naver.com (S.-J.W.); vanthachoub@gmail.com (V.C.); suin917@naver.com (S.-I.C.); juyeolyun9@gmail.com (J.-Y.Y.); 2 Forest Bioinformation Division, National Institute of Forest Science, Suwon 16631, Republic of Korea; iistorm@korea.kr

**Keywords:** fungal/oomycete cell wall, insect cuticle, antifungal and insecticidal activity, cell wall lysis, cuticle degradation, plant pest and disease

## Abstract

Plant diseases and insect pest damage cause tremendous losses in forestry and fruit tree production. Even though chemical pesticides have been effective in the control of plant diseases and insect pests for several decades, they are increasingly becoming undesirable due to their toxic residues that affect human life, animals, and the environment, as well as the growing challenge of pesticide resistance. In this study, we review the potential of hydrolytic enzymes from *Bacillus* species such as chitinases, β-1,3-glucanases, proteases, lipases, amylases, and cellulases in the biological control of phytopathogens and insect pests, which could be a more sustainable alternative to chemical pesticides. This study highlights the application potential of the hydrolytic enzymes from different *Bacillus* sp. as effective biocontrol alternatives against phytopathogens/insect pests through the degradation of cell wall/insect cuticles, which are mainly composed of structural polysaccharides like chitins, β-glucans, glycoproteins, and lipids. This study demonstrates the prospects for applying hydrolytic enzymes from *Bacillus* sp. as effective biopesticides in forest and fruit tree production, their mode of biocidal activity and dual antimicrobial/insecticidal potential, which indicates a great prospect for the simultaneous biocontrol of pests/diseases. Further research should focus on optimizing the production of hydrolytic enzymes, and the antimicrobial/insecticidal synergism of different *Bacillus* sp. which could facilitate the simultaneous biocontrol of pests and diseases in forest and fruit tree production.

## 1. Introduction

Plant diseases and insect pest damage cause tremendous losses in forest plantations and fruit tree production worldwide, by lowering the yield and quality of wood and fruit products, which hurts the livelihood of farmers by reducing the economic returns and impedes the afforestation efforts by lowering the survival of seedlings [1,2]. The use of chemical pesticides, especially synthetic fungicides and insecticides, has been the main strategy for controlling plant fungal diseases and insect pests in nurseries, orchards, and forest plantations for decades [3,4,5]. Most commercial farmers have adopted a routine pesticide application program to combat losses from different diseases and insect pests in nurseries, orchards, forest plantations, and post-harvest [6,7,8,9]. However, the continuous use of chemical pesticides not only poses health and environmental risks, but the sub-lethal exposure of these chemicals has also led to an increasing challenge of pesticide resistance in various insect pests and phytopathogens of economic importance [10,11,12]. The increasing awareness of such previously underestimated health and environmental risks caused by the continuous application of pesticides has stimulated a strong consumer-based demand for eco-friendly and safer alternatives to chemical pesticides [13,14,15].

The use of biological control agents (BCAs) as an alternative to the detrimental use of chemical pesticides has attracted tremendous scientific interest as an environmentally friendly strategy of controlling plant diseases and insect pest damage in agriculture, forestry, and fruit tree production [16,17,18]. Most chemical pesticides often cause environmental contamination and affect non-target organisms [19,20]. Other biological pesticide and disease management strategies such as the use of fungal BCAs could also pose some level of biological risks because they have been proved to spread, stabilize, and alter the soil microbiota [21]. Interestingly, bacterial BCAs have been proved to be both non-toxic and can quickly drop back to natural (environmental) levels shortly after application because of the limited nutrient supply [22], and the biological buffering of the environment [23]. Thus, bacterial BCAs are increasingly becoming favorable alternatives to chemical pesticides [16,24,25,26]. Consequently, various bacterial species including *Bacillus* sp., *Pseudomonas* sp., *Streptomyces* sp., *Lysobacter* sp., and *Seratia* sp. have been studied for antimicrobial and entomopathogenic potential against various plant diseases and insect pests [16,27,28,29,30,31]. Especially, *Bacillus* sp. have been demonstrated to produce metabolites that facilitate their survival, competition, niche colonization, phytopathogenic antagonism and entomopathogenic effects [28,32,33]. Various *Bacillus* sp. have been reported to produce a wide range of lipopeptides [34,35,36], polyketides [37,38,39], bacterial volatile compounds, and other environmental signaling compounds [40,41,42]. Some *Bacillus* sp., especially *B. thuringiensis* (Bt) have also been widely reported and reviewed to produce protein toxins, which are the most widely commercialized biocontrol products worldwide [43,44,45,46,47,48,49]. Except for Bt toxins, the application of most other bacterial control agents has been limited to special high-value crops and greenhouse production, largely due to the high costs of production [50]. Since most antimicrobial peptides from bacterial control agents are often secreted in lower concentrations during the fermentation process, their successful commercialization would require the use of complex extraction, purification, formulation, and packaging techniques for their effective applications at high concentrations [27,50,51]. These expensive processes, along with the high cost of commercial fermentation media still render the application of these antimicrobial peptides practically less feasible, especially in small farms with limited capital investments [27]. Moreover, even the application of *Bt* is currently less effective since many insect pests have developed a resistance to these protein toxins, including resistance against some Bt-transgenic plants [47]. Interestingly, several other *Bacillus* sp. have also been reported to demonstrate phytopathogenic antagonism and entomopathogenic effects against various insect pests through their prolific production of hydrolytic enzymes [40,52,53,54,55]. Unlike antimicrobial peptides, hydrolytic enzymes from *Bacillus* sp. are produced in high concentration during the fermentation process, are highly effective against both phytopathogens and insect pests, and the production of spores can be achieved using locally formulated, cost-effective media and require minimal processing [27,56,57,58,59]. Moreover, *Bacillus* species have unique ecological adaptability for survival under a wide range of environmental conditions, which include the production spores (resistant to a wide range of temperature and pH), prolific reproduction, effective colonization and competition that makes them more favorable for field application than other BCAs [28]. The production of spores by *Bacillus* sp. is also a highly desirable attribute that facilitates the processing, handling, and distribution of the biocontrol products in spore form, which can easily germinate, reproduce, and secrete the hydrolytic enzymes upon inoculation [55,60]. However, despite the increasing research attention about the role of hydrolytic enzymes from *Bacillus* sp. in crop protection, there is still limited and unconsolidated knowledge of their potential applications in the field of forestry and fruit tree production. 

The purpose of this review is to explore the potential application of hydrolytic enzymes from *Bacillus* species in the biological control of phytopathogenic fungi, insect pests and plant parasitic nematodes in forest and fruit trees, from nursery seedlings production to field application. This review provides insights into the prospect for the simultaneous biocontrol of plant diseases/insect pest through the application of hydrolytic enzymes produced by *Bacillus* sp. through the degradation of phytopathogenic cell wall/insect cuticles and provide insights for future research advancements for their optimization and utilization. 

## 2. Hydrolytic Enzymes in Biological Control

### 2.1. The Bio-Fungicide Role of Hydrolytic Enzymes 

**Figure 1:**
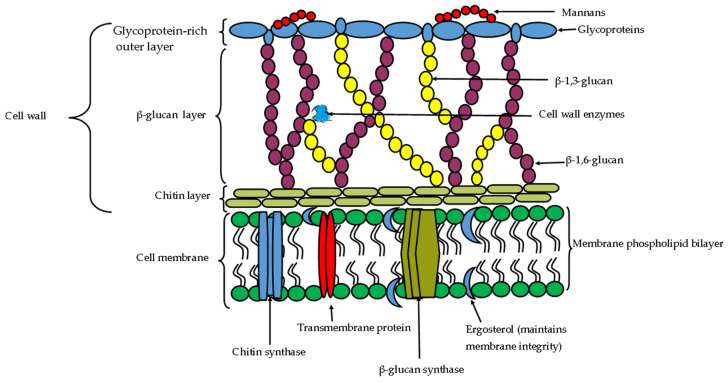
The schematic overview of fungal cell wall (showing the interlinking of chitin, β-glucan, and structural proteins) and the cell membrane phospholipid bilayer organization.

The cell walls of fungal/oomycete phytopathogens are composed of a strong dynamic but flexible matrix of diverse components of embedded and linked polysaccharides (Figure 1), such as amino polysaccharides, glucans (α- and β-glucans), proteins, lipids, and cellulose (in the case of oomycetes), and other important but less prominent components such as melanins, hydrophobins, sporopollenin, and uronic acids [61,62,63,64]. The fungal cell wall composition has structural and functional properties that facilitate fungal interaction with the environment. For instance, fibrous and gel-like carbohydrate polymers (mainly α-chitins (cellulose in oomycetes) which are covalently attached to branched β-(1,3) glucans) form the core (building blocks) of the cell wall and provide tensile strength (to overcome internal hydrostatic pressure) and protection from stress and external aggression [61,62,64,65]. While the diverse interlinking protein polymers (mainly glycoproteins and manmans) and other components, like melanin, glycerol (especially in appressoria) and lipids (such as ergosterol), link to the carbohydrate core to modify the cell wall to facilitate flexibility, selective permeability, surface attachment, spore germination, germ tube and appressoria formation, sporulation, and specialized protection by disguising the cells from phagocytes [61,62,65].

Despite their complexity and the disguised nature of their biosynthetic pathways, numerous research studies have recently revealed that these cell wall components are suitable targets for antifungal agents, especially biofungicide products for the eco-friendly management of phytopathogenic fungi/oomycetes [51,54,55,62,66,67,68,69,70]. Hence, hydrolytic enzymes such as chitinase, β-glucanase, protease, lipase, chitosanases, and cellulase from *Bacillus* sp., have potential applications in the biological control of phytopathogens and insect pests in forestry and fruit tree production [51,53,54,60,71,72,73]. These enzymes hydrolyze specific cell wall components (by breaking the glycosidic linkages that bind the cell wall structural polymers), which then causes the disintegration of cell wall matrices, consequently leading to the loss of protective and functional properties such as selective permeability, tensile strength and turgor-driven cell expansion for growth and host infection [25,74,75].

### 2.2. The Entomopathogenic Role of Hydrolytic Enzymes

**Figure 2:**
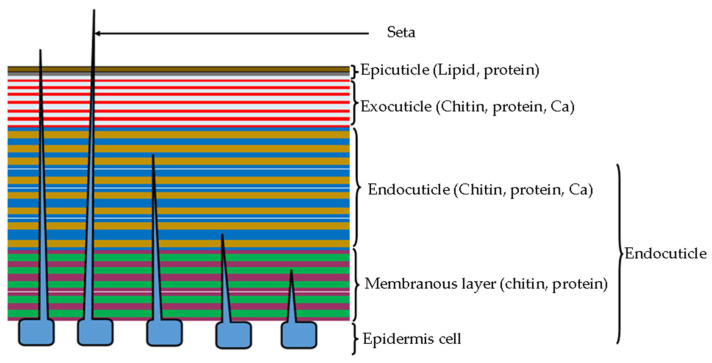
The schematic illustration of insect cuticle with layers of lipid, chitin and structural proteins and other components.

Just like the cell walls of phytopathogenic fungi and oomycetes, the cuticles (epicuticle, exocuticle, and endocuticles) in the exoskeleton and the peritrophic membrane of the hindgut in insect pests are majorly composed of fibrous chitin, with β-1,4 links that transform it into a straight, ribbon-like layer of highly crystalline structure (Figure 2). These chitin fibrils contain sugar residues that are heavily H-bonded, making stiff and chemically stable structures [76]. The fibrous chitin layers are interlinked with diverse glycoproteins through H-bonds which are hardened/sclerotized during growth via the addition of metals and minerals, and deposits of lipid polymers and other components like catechol that are secreted by the epidermal cells [76,77]. The exoskeleton cuticle is often covered by cement and wax deposits to provide another protective layer against desiccation, infection, predation, and provides communication signals [77,78,79,80,81]. The other cuticle components in the appendages and internal organs (hindgut, foregut, and tracheae) strike a balance between mechanical strength (stiffness) and flexibility to facilitate life processes such as respiration, locomotion and flight, and internal protection [77,78,79,80,81]. Thus, the degradation of cuticular chitin nanofibers, protein, and lipid polymers in the cuticle matrix by hydrolytic enzymes from entomopathogens causes devastating effects on insect health, including loss of ecological fitness and mortality, which ultimately reduces the herbivorous activity of insect pests on crops and trees [30,60,72,73,82,83].

## 3. The Major Lytic Enzymes and Their Antimicrobial and Insecticidal Activity

### 3.1. The Prospect of Chitinases as Antifungal and Insecticidal Agents

**Figure 3:**
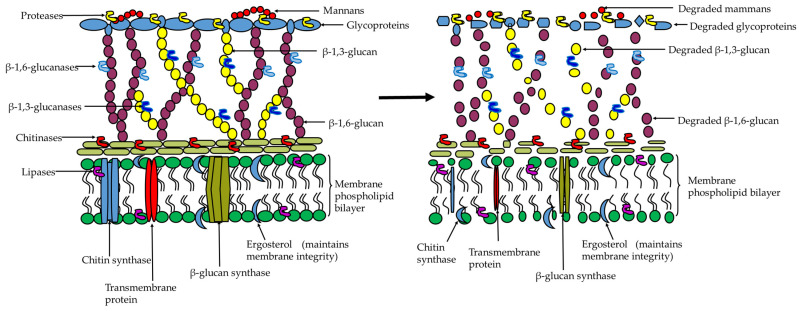
The illustration of fungal cell wall degradation by hydrolytic enzymes secreted by *Bacillus* sp.

Chitinases hydrolyze the β-(1,4)-glycosidic linkage of the chitin polymer in the fungal cell walls and insect cuticles (mainly endo- and exo-cuticles) to release N-acetyl-d-glucosamine (GlcNAc) and chitooligosaccharides units [84]. The mechanism of chitinolytic activity is achieved by endo-chitinases which cleave chitin polymers into the respective oligomers, exo-chitinase which catalyzes the breakdown of β-1,4-glycosidic bonds between the 2-GIcNAc and 3-GIcNAc residues to release chitobiose, and N-acetylglucosaminidase (NAGase) which cleaves the β-1,4-glycosidic bond at the end of the chitin chain to release GlcNAc [85,86,87]. Sometimes, these enzymes overlap with the activity of chitosanases which hydrolyze both chitosan (GlcN-GlcN linkages) and β-1,3- and β-1,4-glucans [85,86,87]. Thus, the degradation of cuticular chitin by chitinases from *Bacillus* sp. causes lethal effects (Figure 3), and loss of ecological fitness in surviving organisms. For instance, chitinase from *B. thuringiensis* strain NM101-19 and *B. licheniformis* strain NM120-17 demonstrated a potential to degrade the cell wall of various soyabean fungal phytopathogens including *Aspergillus* sp., *F. oxysporum*, *P. chrysogenum, Pythium* sp., *R. solanacearum*, *Verticillium* sp., *Rhizoctonia* sp., *Rhizopus* sp., and *Trichoderma* sp., which consequently improved seed germination [70]. The chitinase (31 kDa) from *B. subtilis* strain NPU 001 and chitinase (48 kDa) from *B. cereus* strain YQ308 grown on shrimp and crab shell powder (as carbon source) caused effective inhibition against the mycelial growth of *F. oxysporum* and *P. ultimum*, respectively, at a concentration of 2 mg/mL [57,58]. Similarly, chitinase from *B. thuringiensis* var *israelensis* grown on shrimp waste effectively inhibited the growth of *S. rolfsii,* (that causes southern blight in soybean) and other phytopathogens such as *Rhizopus* sp., *Fusarium* sp., and *Aspergillus* sp. (in vitro), and consequently increased soybean seed germination in a dose-dependent manner, from 0.2 to 0.8 units of chitinase/mg of protein [88]. Numerous other examples of the potential role of chitinases have been demonstrated in various studies. For stance, ChiCW and ChiCH from *B. cereus* strain 28-9 inhibited the conidial germination of *B. elliptica* that causes botrytis leaf blight of lily by 84% and reduced the disease severity on detached leaves [89]. Antifungal chitinase ChiIO8 from *Bacillus cereus* IO8 and ChiS and ChiL from *B. pumilus* strain SG2 effectively inhibited the mycelial growth of several phytopathogenic fungi including *Botrytis cinerea, R. solani* (blight diseases), *Verticillium* sp., (Verticillium wilt), and *Stemphylium botryosum* (Stemphylium blight), but did not inhibit the oomycete pathogens *P. citricola* and *P. capsici* since their cell walls are composed of cellulose instead of chitin polymers [90,91]. Extracellular chitinase (55 kDa) produced by *B. licheniformis* strain MY75 in the presence of chitin substrate demonstrated complete in vitro inhibition of spore germination and subsequent mycelial growth of *Gibberella saubinetii* (known as the perfect stage of *F. graminearum* that causes head blight in cereals) and *A. niger* (black mold in fruits) [92]. A thermotolerant (50 °C) chitinase (30 kDa) from *B. subtilis* strain SL-13 showed effective mycelial growth inhibition of *R. solani* by causing severe hyphae disintegration (lysis) and protoplast leakage within 48 h, and consequently reduced tomato wilt disease, increased seed germination and seedling growth under greenhouse conditions [93]. Chitinases FI (24 kDa) and FII (16 kDa) from the *B. amyloliquefaciens* strain V656 showed specific chitinase (and the corresponding EC_50_ against *F. oxysporum*) activity of 0.34 (and 4.69) and 0.11 (and 3.16) units/mg, respectively [56]. Extracellular chitinase (43.7 kDa) from *B. cereus* strain NK91 demonstrated a dose-dependent response and inhibited the mycelial growth of *F. oxysporum* (66.7%), *R. solani* (64.6%), and *C. gloeosporioides* (63%) [94], while chitinase from *B. licheniformis* strain J24 inhibited the growth of *F. pseudograminearum* and reduced the severity of *Fusarium* rot on corn seeds [95]. Chitinase from *B. subtilis* strain TV-125, *B. subtilis*, *Bacillus* sp. strain 739, and *B. pumilus* RST25 inhibited *F. culmorum* (root rot in vegetables) [96], *Aspergillus* sp. and *P. chrysogenum* [97], *Fusarium* sp. and *Helminthosporium sativum* [98], and *F. solani* and *A. niger* in *Triticum aestivum* [99], respectively. The chitinase from *B. chitinolyticus* (SGE2, SGE4, and SSL3), and *B. ehimensis* (MG1) also exhibited antifungal activity against *A. nidulans*, *B. cinerea*, *F. culmorum*, *S. sclerotiorum* and *Guignardia bidwellii* [100], and chitinase from *B. licheniformis* strain TCCC10016 was effective against *F. oxysporum* [101], while chitinase from *B. pumilus* strain CCIBP-C5 antagonized *Pseudocercospora fijiensis* Morelet that causes black sigatoka disease in banana [102]. Exochitinase, ChiA (49 kDa) and endochitinase and exochitinase, ChiB (80 kDa) inhibited the growth and conidial germination of *F. verticillioides*, a major cause of rot and wilt diseases in maize [103]. Chitinase (65 kDa) from *B. licheniformis* strain ATCC 14580 antagonized several phytopathogens including *Phoma medicaginis* that causes damping-off disease in *Medicago truncatula* and effectively reduced the diseases symptoms [104], while chitinase from *B. licheniformis* strain PR2 caused hyphal alterations and inhibited the growth of *B. cinerea* and *C. gloeosporioides* and consequently reduced fruit rot diseases in jujube [105]. 

Moreover, several studies have also reported the synergism of chitinase with other enzymes such as protease and β-l,3-glucanase to exert stronger antimicrobial activity. For example, *B. velezensis* strain CE 100 has been reported to produce chitinase along with protease and β-l,3-glucanase, which together inhibited the growth and spore germination of *C. gloeosporioides* and controlled walnut anthracnose disease at the same rate as chemical fungicide [51]. Chitinase, β-l,3-glucanase, and protease from *B. velezensis* strain CE 100 were reported to control *Pestalotiopsis maculans* (leaf blight disease in *Quercus acutissima* Curruth) and substantially increased the survival of seedling [53], and *Macrophomina phaseolina* and *F. oxysporum* f. sp. *fragariae* (charcoal rot and fusarium wilt diseases in strawberry, respectively) [55]. Chitinase and β-1,3-glucanase from the *B. licheniformis* strain MH48 also controlled *F. oxysporum* (*Fusarium* root rot in coastal pine seedlings in forest nurseries) [106], and *B. cinerea, Glomerella cingulata*, *Pestalotia diospyri*, and *P. karstenii* (foliar fungal diseases of *Camellia oleifera* seedling), and improved the seedling quality [107]. 

**Figure 4:**
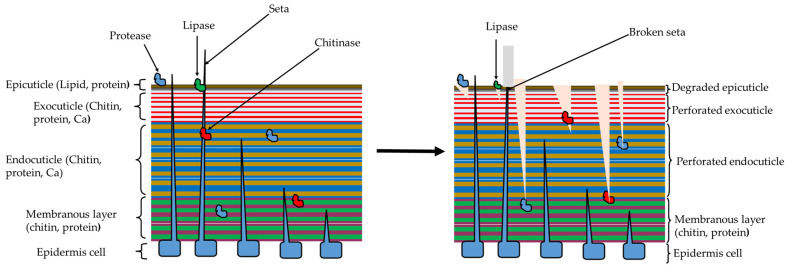
The illustration of the insect cuticle degradation by hydrolytic enzymes from *Bacillus* sp.

Similarly, the chitinases have been widely reported to hydrolyze the chitin fibrils in insect cuticle exoskeletons and gut lining to cause toxicity and subsequent insect mortality to protect plants from herbivorous activity of these phytophagous insects (Figure 4). For example, chitinase from *B. cereus* strain 1.21 isolated from soil rhizosphere was reported to degrade chitin polymers in the exoskeleton cuticle of *Bemisia tabaci Genn.* (Hemiptera: Aleyrodidae), a notorious sap-sacking insect pest of numerous crops such as chili pepper [108]. Chitinase from *B. licheniformis* strain USMW10IK, an endosymbiont from *Globitermes sulphureus* worker termite, demonstrated an effective termiticidal activity within 24 h when in contact with termite exoskeleton of the same termite species (topical application), but higher concentrations would be required to cause termite mortality in the soil environment [109]. Recently, the combined activity of crude fractions of chitinase and protease from *B. velezensis* strain CE 100 [72], and *B. licheniformis* strain PR2 [60] demonstrated an effective termiticidal activity against subterranean termite, *Reticulitermes speratus kyushuensis* Morimoto (Isoptera: Rhinotermitidae), through the degradation of exoskeleton cuticle. Both studies provided insights into the prospect of using these *Bacillus* sp. as entomopathogens in protecting precious wooden architectural buildings of cultural heritage worldwide, as well as high value susceptible trees such as *Pinus densiflora* Siebold and Zucc. (Pinales: Pinaceae) against termite damage [60,72]. In a separate study, chitinase and protease from entomopathogenic *B. velezensis* strain CE 100 demonstrated effective degradation of the exoskeleton cuticle of *Dasineura jujubifolia* Jiao and Bu, sp. Nov. (Diptera: Cecidomyiidae) larvae (jujube gall midges), and suppressed pest infestation/leaf damage in the jujube orchard [73]. Chitinase and proteases from *B. licheniformis* PR2 have also been reported to degrade the cuticular polymers in the exoskeleton of *Hyphantria cunea* Drury (Lepidoptera: Erebidae) larvae that causes massive defoliation of high-value bioenergy forest trees of *Populus × canadensis* Moench [71]. Based on histological examination, treatment with chitinase and protease enzymes causes a wide range of effects from partial lacerations on the epicuticle, damage/deformations of the peripheral organs such as bristles, sensilla and sockets, to severe disintegration of the cuticle [60,71,72,73]. Chitinase (25 kDa) from *B. cereus* strain C-13 when treated on the leaves at a concentration of 0.048 U/mL caused 78% mortality against adult *Helopeltis theivora* Waterhouse (Hemiptera: Miridae), the sap-sacking tea mosquito bugs that cause tremendous damage in *C. sinensis* (L.) O. Kuntze plantations in India [110]. Based on the mode of application, the chitinase from *B. cerus* strain C-13 could have affected the cuticular chitin in the gut of *H. theivora* since the treated leaves were air-dried for 30 min before introducing the insects.

Chitinases from *Bacillus* species have also demonstrated direct insecticidal activity or enhanced the insecticidal activity of already known insecticidal toxins in model insects that are not necessarily phytophagous but could provide the basis for evaluating the chitinolytic potential of these bacterial strains. For example, exochitinase (66 kDa) from *B. thuringiensis* subsp. *pakistani* caused a toxicity of up to 70.0% mortality against *Aedes aegypti* larvae at 64 mU/mL [111]. The chitinolytic enzyme *B. atrophaeus* strain A7 inhibited the development of *Drosophila melanogaster* larvae into adults (LD_50_ = 17.3 ± 1.4 mU/mL), which further demonstrates the insecticidal potential of chitinases from *Bacillus* species [112]. Even though *A. aegypti* and *D. melanogaster* are not phytophagous insects, the biocontrol activity of chitinases against these insects indicates the potential to control some of the notorious sap-sacking insects pests, either as the direct cause of insecticidal activity or as a factor that weakens the gut cuticle layer to facilitate the pathogenicity of other toxins.

Chitinases have been demonstrated to alter the physiological and metabolic processes of insect pests, thus indirectly exerting insecticidal activity. For example, the two extracellular chitinases (CS1 and CS2) produced by *B. subtilis* demonstrated insecticidal activity against *Spodoptera litura* Fab. (Lepidoptera: Noctuidae) larvae by degrading the gut cuticle in the peritrophic membrane and epithelial cells (based on histological analysis) and by decreasing the larvae gut enzymes lactate dehydrogenase, acid phosphatase, alkaline phosphatase, and adenosine triphosphatase which are vital for normal insect metabolic activities [113,114]. Moreover, Chandrasekaran et al. (2012) also demonstrated that besides causing insect mortality, the larvae that were fed on leaves contaminated with chitinase had a substantially lower rate of growth and reduced body weight, which could indicate a loss of ecological fitness in surviving insects that may survive the direct lethal effect of chitinase. In another study, the chitinolytic activities of exochitinases from *B. thuringiensis* subsp. *israelensis* strain IPS78 and *B. thuringiensis* subsp. *aizawai* strain HD133 were also reported to play a role in the bacterial pathogenesis against the host insects: *Culicoides nubeculosus* (Diptera: Ceratopogonidae) larvae (midges) and *Spodoptera littoralis* (Lepidoptera: Noctuidae), the caterpillars of cotton leafworm, respectively [115]. Since these *B. thuringiensis* strains are well known to possess highly specific insecticidal toxins, it is most probable that the role of these chitinases was to degrade or weaken the peritrophic membranes to pave way for the attachment of other toxins and the inhibition of these exochitinases (using 100 μM allosamidin) caused a considerable drop in the insecticidal activity of both entomopathogens [115,116]. Similarly, a recombinant chitinase (36 kDa) from *B. thuringiensis* strain HD-1 also demonstrated the potential to enhance the insecticidal effect of the vegetative insecticidal protein (Vip) against neonate larvae of *S. litura,* potentially via the weakening of the gut cuticle to increase the attachment of Vips [117]. Thus, the role of chitinases in enhancing the pathogenesis of insecticidal toxins from *B. thuringiensis* and baculovirus was detailed, where they cause hydrolysis of cuticle polymers in the gut to facilitate toxin penetrations or act as adjuvants, and thereby increase the efficacy of insecticidal activity as evidenced by the reduced lethal time ad higher mortality rate [118,119]. When the chitinase gene was transferred from *B. subtilis*, a chitinolytic entomopathogen with high toxicity against *Aphis gossypii* (Hemiptera: Aphididae), into several strains of *B. thuringiensis* that were more effective against *Tuta absoluta* Meyrick (Lepidoptera: Gelechiidae), the two of the recombinants Tr5 and Tr10 showed a strong chitinolytic activity, exerting a stronger insecticidal effect than their parental strains [120]. This demonstrates that the chitinolytic activity of *Bacillus* strains has a synergic potential on the pathogenicity of insecticidal toxins secreted by antipathogenic *B. thuringiensis* strains. Thus, besides targeting cuticle exoskeleton degradation, purified chitinases from *Bacillus* sp., or the bacterial filtrate or crude enzyme fraction containing chitinase could enhance the insecticidal activity of other toxins from well-known entomopathogens such as Bt. The synergy of chitinase and such insecticidal toxins should be further studied and optimized to achieve the highest entomopathogenic effect under field conditions against susceptible insect pests.

### 3.2. The Prospect of Proteases from Bacillus sp. as Antifungal and Insecticidal/Nematocidal Agents

The fungal cell wall is composed of a glycoprotein outer layer while the epicuticle and exocuticle are interlinked with other structural polymers (Figure 1). These structural protein components are a suitable target for the hydrolytic activity of proteases into small peptides, which lead cell lysis and cellular leakage [51,53,54]. The antimicrobial activity of proteases against phytopathogens has been widely reported over the years and is mainly premised on the wide distribution of structural and functional proteins (such as membrane proteins) of the target pathogen such as the mannoproteins, glycoproteins in the hyphal cell walls, and their role in conidial germination, cell attachment and appressorial formation and environmental interactions [63,65,121]. Thus, the degradation of the fungal cell proteins has been reported to play a vital role in fungicidal and fungistatic activities, specifically in the bacterial (*Bacillus* sp.) antagonism of phytopathogenic fungi. For instance, the serine proteases (44.3 kDa) from *B. licheniformis* strain W10 and *B. licheniformis* strain TG116 demonstrated antifungal activity against *B. cinerea* [122], and against *P. capsica, R. solani, Fusarium* sp., and *B. cinerea* [123], respectively. The protease from *B. subtilis* strain 21 was effective against *F. verticillioides* and *R. solani* in strawberry plants under greenhouse conditions [124], while the protease (31 kDa) from *B. licheniformis* strain BS-3 was effective against *A. niger, M. oryzae, R. solani*, and *F. oxysporum* strains [125]. The combined effect of protease, chitinase, and β-1,3-glucanase from *B. velezensis* inhibited spore germination, germ tube elongation and mycelial growth of *C. gloeosporioides* (anthracnose disease in walnut) [51] and controlled the mycelial growth of *Phytophthora* sp. (*Phytophthora* root rot/wilt) [54].

Similarly, proteases are a major component of the endocuticle, and the membranous layers of the insect exoskeleton contain a substantial composition of structural proteins, interlinked with other structural polymers such as chitin (Figure 2), making it a suitable target for the proteolytic activity. The proteolysis of cuticular proteins to peptides causes cuticle degradation and exposure of internal tissue to environmental aggression [30,60,71,72,73]. The insecticidal role of proteases, mainly cysteine proteases, insect-toxic metalloproteases and serine proteases from *Bacillus* sp. have been reported to control insect pests and plant parasitic and free-living nematodes of potential economic importance in forestry and fruit tree production [126]. For example, *B. velezensis* strain CE 100 and *B. licheniformis* strain PR2 produced a combination of protease and chitinase that degraded the exoskeleton cuticles of several insect pests including *D. jujubifolia* [73], *H. cunea* [71] and *R. speratus kyushuensis* [60,72]. The proteolytic degradation of insect exoskeleton and gut cuticles is due to the fact the cuticle matrix is mainly composed of glycoproteins and proteoglycans (cuticular protein polymers) which can be hydrolyzed by proteases into simple peptides and amino acids, leading to disruption and loss of structural and function properties of the cuticles [60,71,72,73]. Even though most earlier studies had focused on the proteolytic activity of fungal entomopathogens [83,127], more recent studies have demonstrated a similar protease activity from entomopathogenic bacteria [128,129,130]. The major consensus about the mode of insecticidal activity in both the bacterial and fungal entomopathogens is that the proteolytic activity substantially enhances the chitinolytic degradation of the cuticle since the hydrolysis of the cuticular proteins (that shield/ embeds the chitin fibers) exposes the cuticular chitin fibrils, which increases the surface area for chitinase activity [73,83]. Moreover, proteases have been demonstrated to perforate the basement membranes through the hydrolysis of cuticular protein, which then facilitates the pathogenesis of other insecticidal protein toxins as demonstrated by *Autographa californica* nucleopolyhedrovirus (AcMNPV) against *Heliothis virescens* [131]. Proteases from entomopathogenic *Bacillus* sp. have also been identified as pathogenic factors for the nematocidal proteins, where they hydrolyze the host cuticle to facilitate the pathogenesis of other toxins or cause direct mortality by disrupting the cuticle to cause lethal physiological alterations [132]. For example, *Bacillus* sp. RH219 produced cuticle-degrading protease Apr219 (33 kDa at 930 U/mL) which degraded the cuticle of *Panagrellus redivivus* nematodes and caused 97% mortality within 48 h while a neutral protease Npr219 (41 kDa, at 870 U/mL) from the same strain only caused 20% mortality [132]. The combination of both protease enzymes increased the nematocidal activity of protease Apr219 by 9%, indicating some level of synergism [132]. Extracellular alkaline protease *BLG4* gene was demonstrated to produce the major nematocidal protein of the entomopathogenic bacterium, *Brevibacillus laterosporus* strain G4 and effectively destroyed the cuticle of *P. redivivus* and by adding another neutral protease NPE-4 from *B. subtilis*, the nematocidal activity of *B. laterosporus* strain G4 was further enhanced, demonstrating the insecticidal synergism of the various proteases [128]. A neutral protease Bae16 (40 kDa) from *B. nematocida* strain B16 degraded the cuticular gelatin and collagen of *P. redivivus* and *Bursaphelenchus xylophilus* (pine wood nematode) and caused nematode mortality after 2 h, with LC_50_ of 1.69 μg/mL and 2.26 μg/mL, respectively [133]. When combined with serine protease, the LC_50_ was improved to 0.99 and 1.40 for *P. redivivus* and *B. xylophilus* nematodes, respectively [133]. Serine protease (28 kDa) from *Bacillus* sp. strain B16 demonstrated nematocidal activity against *P. redivivus* by hydrolyzing native proteinaceous substrates in the nematode cuticle and caused 90% nematode mortality within 24 h [130]. Alkaline serine protease Bace16 and a neutral protease Bae16 from *B. nematocida* strain B16 were independently identified as key virulence factors against *P. redivius* and *B. xylophilus* nematodes, and a genetically overexpressed recombinant strain Bace16 increased the proteolytic and nematocidal activities by 62% and 80%, respectively [134]. Alkaline protease (28 kDa) from endophytic *B. cereus* strain NJSZ-13 isolated from healthy *Pinus elliottii* trunk caused severe cuticle degradation of *B. xylophilus* nematodes and caused complete mortality within 72 h, demonstrating a symbiotic relationship of the bacteria and pine tree [135]. Moreover, based on the simple structure of nematode eggshells (containing chitinous and lipid layers), entomopathogens with the potential to produce both proteases, chitinase and lipase could effectively degrade nematode cells and suppress the density of nematode juveniles and plant infestation [16,136]. This represents one of the most promising strategies for controlling plant nematodes in forest and fruit tree production that could be utilized for the effective and environmentally friendly control of nematode infestation.

### 3.3. The Prospect of β-Glucanases from Bacillus sp. in Biological Control

The β-glucans form a major structural component of the fungal cell wall, linking up the mannoproteins/glycoproteins to chitin fibrils. They are vital for the structural strength and shape of the cells and withstanding turgor pressure in the hyphal and appressorial cells. The mechanism of antifungal/anti-oomycete activity of β-1,3-glucanasese and β-1,6-glucanasese is based on the hydrolysis of the glucosidic linkages of β-1,3-glucans and β-1,6-glucans in the fungal/oomycete cell to produce glucose monomers, leading to the degradation of vital structural components of the fungal cell wall [53,54,55]. The hydrolysis of β-glucans by β-glucanasese from *Bacillus* sp. has been proposed as an effective fungicidal strategy [63,65,66,74]. For instance, β-1,3-glucanase from *B. velezensis* strain CE 100, along with chitinase and protease, causes an antagonistic effect against several phytopathogens such as *C. gloeosporioides* (walnut anthracnose) [51], *P. maculans* (leaf blight in oak seedlings) [53], *M. phaseolina* and *F. oxysporum f.* sp. *fragariae* (charcoal rot and *Fusarium* wilt in strawberry) [55], and against *Phytophthora* sp. (root diseases in Japanese cypress seedlings) [54]. The β-1,3-glucanase from *B. licheniformis* strain MH48 antagonized *F. oxysporum* (*Fusarium* wilt coastal pine seedlings) [106], and *B. cinerea*, *G. cingulata*, *P. diospyri*, and *P. karstenii* (foliar diseases in *C. oleifera* seedling) [107]. The β-1,3- and -1,4-glucanase (27.3 kDa at 1706 U/mL) from the *B. velezensi*s strain ZJ20 caused cell wall lysis of the mycelia against *Cryphonectria parasitica*, *Helicobasidium purpureum*, and *Cylindrocladium quinqueseptatu*m [137], while the β-glucanase (10 kDa) from *B. subtilis* strain CW14 inhibited the mycelial growth and spore germination of *A. ochraceus*, and its recombinant reduced fungal infection in soybean by 96% [138]. The β-1,3-glucanase (40 kDa) from *B. amyloliquefaciens* strain MET0908 inhibited *C. lagenarium* (watermelon anthracnose) [139], and β-1,3-glucanase from *B. subtilis* strain NSRS 89-24 inhibited *Pyricularia grisea* (rice blast) and *R. solani* (rice sheath blight) [140]. Based on the molecular and biochemical studies, β-1,3-glucanase from *Bacillus* sp. mainly enhances the antifungal activity of other hydrolytic enzymes such as chitinases which are often produced in higher concentrations by most biocontrol strains [51,106], or they could even supplement the role other antimicrobial lipopeptides like fengycin, surfactins and iturin [141]. This is because β-glucans are structurally embedded between proteins and chitin polymers; thus, their degradation by contact antifungal molecules such as hydrolytic enzymes is expected to be relatively lower compared to chitin and protein hydrolysis. Their hydrolysis could be enhanced by the combined effect of protease or/and chitinase, which hydrolyzes the embedded structural polymers to increase the surface area for β-glucanase activity [53,54,55].

### 3.4. The Role of Lipases from Bacillus sp. in Biological Control

Lipids are quite minor components of the fungal cell wall, and their specific composition and abundance could have a different distribution depending on species and phase of growth, and are important components in the vegetative and reproductive phases, such as the sporidismolides [61]. Their specific role has not yet been well studied but could include the protection of spores from bacterial invasion and preventing desiccation [61]. Lipases are members of a hydrolase enzyme category that is abundantly distributed in nature, composed of GX1SX2G active site, where G, S, X1, and X2 represent glycine, serine, histidine, and aspartate or glutamate residues, respectively [142]. They catalyze the hydrolysis of lipoids (including fats, waxes, sterols, glycerides, carboxylic acid esters, etc.), usually at the interface of aqueous/organic phase of various structural components [142]. 

There are some examples where the different forms of lipases have been reported for antimicrobial activity against some important phytopathogens, such as the mycolytic activity of *Bacillus* sp. strain 739 against *Bipolaris sorokiniana* (cereal root-rot), but the activity of lipase was mainly supplemental to the hydrolytic activity of chitinases, proteases, and β-1,3-glucanases [143]. Lipase (62 kDa) from *Bacillus* sp. strain X-b, along with other hydrolytic enzymes (chitinases, and chitosanase) was enhanced in the presence of fungal cell substrate (0.5%), which indicates its antifungal potential [144], while lipase from *B. subtilis* strains AI01 and AI03 showed antifungal properties against several phytopathogenic fungi, especially *F. solani* (*Fusarium* wilt disease in egg plants) [145]. However, the structure of most fungal pathogens is more complex, and the singular activity of lipases could be insufficient to control fungal pathogens. Nonetheless, lipases could substantially increase the efficacy of other hydrolytic enzymes such as proteases, chitinases and β-1,3-glucanase by hydrolyzing the surface lipids to increase the surface contact of other enzymes to their specific structural substrates in the fungal cell wall.

Similarly, lipases can hydrolyze the lipoproteins, waxes and fats present in the insect integuments and compromise cuticle protection in insect pests and consequently expose the cuticular chitin and proteins to further degradation by chitinase and proteases, which causes insect mortality by desiccation due to the removal of the waterproof wax layer and exposure of internal organs to external aggressions [26]. For instance, lipases from *B. subtilis* strain Ehrenberg hydrolyzed the wax layer in the cuticle of *Planococcus citri* (Hemiptera: Pseudococcidae), causing lethal effect and significantly reducing female longevity, fecundity, and adult formation in the surviving citrus mealybugs [146]. Lipase activity from *B. subtilis*, along with other hydrolytic enzymes, was reported to degrade the wax and other structural polymers of *Maconellicoccus hirsutus* (Hemiptera: Pseudococcidae), which caused pink mealybug mortality and substantially reduced the ecological fitness of surviving insects by lowering the longevity, fecundity, and body weight, and reducing the wax, sugars, and proteins in the secreted honeydew [147]. The consortium of biocontrol bacteria including *B. altitudinis* that produces lipase (along with protease) effectively degraded the lipid microfibril framework of *Alitropus typus* (lsopoda: Aegidae), a notorious parasite of *Oreochromis niloticus* fish, which demonstrates the prospects of lipase from *Bacillus* sp. against parasitic fruit pests which have a similar mode of life [148]. Moreover, other *Bacillus* strains such as *B. cereus* strain WPD are known entomopathogens of important arthropods such as *Litopenaeus vannamei* (shrimp) where they cause white patch disease (WPD), and their lipolytic activity is among the most important virulence factors [149]. Given the similarities in the cuticle composition of most arthropods, the study of Velmurugan et al. (2015) demonstrates the entomopathogenic prospects of lipase-producing *Bacillus* sp. in the biological control of insect pests of economic importance in forestry and fruit tree production.

### 3.5. The Role of Amylases from Bacillus sp. in Biological Control

Amylases are important enzymes that are required to hydrolyze polysaccharides (starch) mainly in the fungal cell walls into simple sugar unites, which contributes to overall cell wall degrading activity by *Bacillus* sp. in biological control fungal (and bacterial) phytopathogens. For example, the amylase produced by *Bacillus* sp. strain KD7 inhibited the growth of *A. flavus* and consequently suppressed aflatoxin production [150]. The amylolytic activity of *B. subtilis* strains GM2 and GM5 were also among the antifungal factors responsible for the inhibition of mycelial growth and spore germination of *Fusarium* sp., which improved the survival rate of wheat seedlings [151]. The amylolytic activity of *B. velezensis* strains HY-3479 has been reported to be among the antifungal mechanisms for the antagonistic effect against the growth of several phytopathogenic, including *C. acutatum*, *Cylindrocarpon destructans*, *R. solani*, and *S. sclerotiorum* [152]. Similarly, the amylolytic activity of *B. licheniformis* was among the antagonist factors against *V. dahlia*, *F. oxysporum*, *Phytophthora* sp., *C. acutatum*, *B. cinerea*, and *Aspergillus* sp. [153]. Amylase production by other *Bacillus* species such as *Bacillus* sp. strain HE613660 has also been reported to contribute to their antibacterial effect against other common pathogens like *Staphylococcus aureus*, *Escherichia coli*, *Klebsiella pneumoniae*, *Streptococcus agalactiae*, and *Proteus mirabilis*, and pathogenic yeast, *Candida albicans*, which demonstrates the prospect of controlling bacterial phytopathogens in forest and fruit tree production [154]. Further research should be dedicated to the explication of the bactericidal potential of amylase-producing *Bacillus* sp., especially in the biocontrol of post-harvest bacterial fruit rot diseases, to explore their efficacy and applicability. 

### 3.6. The Role of Cellulases from Bacillus sp. in Biological Control

Cellulose is a polysaccharide of β-(1,4)-linked D-glucose units ([C_6_H_10_O_5_]_n_, where n is at least 3000 units) that make up the cell wall structure of oomycetes, algae and plants, and is an important target in the biocontrol of phytopathogenic oomycetes based on the concept of cell wall degradation. The mechanism of cellulase in the degradation of the oomycete cell wall, which is mainly composed of cellulose, is based on the hydrolysis of cellulose structural polymers into simple monomers such as pentose and hexose, leading to cell lysis, cell deformation and the leakage of cellular contents [155,156,157,158]. This results in the inhibition of mycelial growth and zoospore germination, which ultimately suppresses the virulence of the phytopathogen [54,156,157]. For instance, *B. subtilis* strain B71 produced cellulase enzymes and effectively antagonized the growth of plant-pathogenic oomycete, *P. spinosium* in a concentration-dependent manner [155]. The cellulase from *B. subtilis* strain EG21 (along with extracellular pectinase and chitinase) inhibited the growth of *P. infestans* (phytophthora blight disease in potato) and consequently lowered zoospore germination and infection [156]. The cellulolytic activity of *B. amyloliquefaciens* strain UQ154 and *B. velezensis* strain UQ156 inhibited the growth of *Phytophthora* sp. by antagonizing hyphal growth and consequently reduced phytopathogenic load and disease severity in infected pepper plants [157]. The cellulase from *B. velezensis* strain 6-5 inhibited *P. infestans* (potato blight disease) by more than 90% [158], while cellulase (along with protease and mannanase) from 13 *B. pumilus* strains inhibited phytopathogenic oomycetes such as *P. ultimum* and *Aphanomyces cochleoides* and inhibited phytopathogenic fungal and bacterial pathogens, depending on the type of growth media [159]. The cellulolytic activity of *B. licheniformis* strain BL06 inhibited the mycelial and sporangial development of *P. capsici* (*Phytophthora* blight of peppers) [160], and cellulase from *B. velezensis* strain SN337 inhibited *P. sojae* (root rot disease in soybean) [161]. However, despite the seemingly obvious mechanism based on the degradation of the cellulose-containing hyphae cell wall of oomycetes, there is need for further research to demonstrate the anti-oomycete role of purified cellulase and their detailed mode of action. The role of cellulase could be further enhance by dual or multiple application with other hydrolytic enzymes such as protease since the cellulose in the oomycete cell wall are interlinked and embedded in the matrix of other structural polymers such glycoproteins. The summary of hydrolytic enzymes from *Bacillus* strains that have been reported for antifungal and insecticidal activity in various plants is presented (Table 1). 

## 4. Prospects for the Practical Application of Lytic Enzymes from *Bacillus* sp. as Alternatives to Chemical Pesticides in Forest and Fruit Tree Production

### 4.1. The Biocontrol Prospect of Hydrolytic Enzymes from Bacillus sp. against Fungal/Oomycete Phytopathogens and Insect Pests

The main challenge in the application of biocontrol technologies includes the high cost of production, which is mainly due to the high cost of fermentation media, the high initial investment of installing expensive fermenters that minimize the risk of contamination with other ubiquitous microbes, and the cost of purifying and concentrating the antimicrobial products to their effective concentrations [27,50,51,165]. The use of cost-effective fermentation media mainly through studying the efficacy of locally available energy sources such as crustacean shells has been previously reported to reduce the cost of producing *Bacillus* sp. by at least 30-fold without compromising their biocontrol efficacy [165]. Singh et al. (2012), studied the optimal conditions for chitinase (20 kDa) production from a rhizobacterium isolated from chickpea (*Lysinibacillus fusiformis* strain B-CM18), in which a medium containing about 6% chitin, 4.5% NaCl and 0.55% starch and yeast extract increased the production by 56.1-fold at 32.5 °C [166]. Lakshmi et al. (2014) also optimized the conditions for protease production from *B.* licheniformis strain MTCC7075, in which the medium contain 3% rice husk as C-source and various combinations of mineral salts, which increase protease production from 98 U/mL to 184 U/mL [167]. Several other studies have reported optimized conditions for amylase [150] and β-glucanase [168] production from different *Bacillus* sp., but there is limited knowledge about the optimal conditions for the holistic production of the different hydrolytic enzyme combinations. Since these enzymes have demonstrated antimicrobial/insecticidal synergism and the prospect for simultaneous biocontrol of phytopathogens and insect pests, there is a need to study the optimal conditions for the holistic production of the hydrolytic enzyme combinations from *Bacillus* sp. and their effective application rates to the maximum efficacy. 

**Figure 5:**
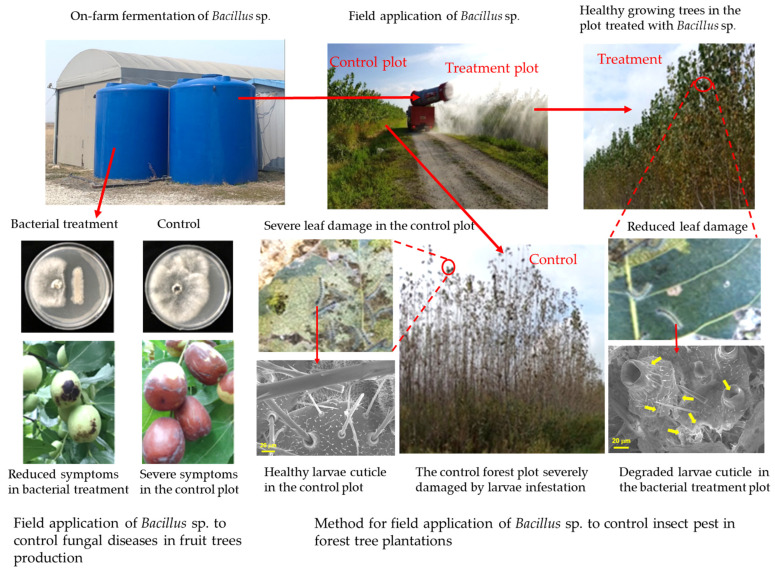
The illustration of field application of hydrolytic enzyme from *Bacillus* sp. in the control of insect pests in high-value biomass forests [30,105].

In addition, among the major advantages for the practical application of the hydrolytic enzymes from *Bacillus* sp. in the biocontrol of fungal/oomycete pathogens and insect pests include their prolific secretion compared to other antimicrobial metabolites such as volatile organic compounds and lipopeptides, which are often produced in smaller concentrations that would require complex purification processes for their effective usage [40,50,51,52,53]. The high production of lytic enzymes from *Bacillus* sp. eliminates the need for costly purification processes since they are produced in effective concentrations in the media which makes their application more practicable [51,53,54,55]. Moreover, the thermophilic nature of *Bacillus* sp. allows effective on-farm fermentation and field application (Figure 5), with minimum risk of contamination since most ubiquitous bacteria cannot flourish under similar conditions [169]. Unlike volatile compounds and antimicrobial peptides that are best applied in closed environments such as greenhouses, lytic enzymes from *Bacillus* sp. do not require complex application techniques, and several studies have demonstrated their effective application against fungal/oomycete infections through seed-dressing [99], and by direct foliar spray [51,53,54,55]. Moreover, several studies have demonstrated a great prospect of producing effective concentrations of lytic enzymes through mass production of *Bacillus* sp. on locally available medium, including the crustacean shells that have high chitin content for enhancing the production of chitinase, and this enhances their practical production and application in large-scale tree nurseries and orchards [57,58,88]. Thus, the hydrolytic enzymes from *Bacillus* sp. hold great prospects in the biological control and integrated pest and plant disease management due to their ease of production, diverse applications against both phytopathogens, insect pests and plant parasitic nematodes, and their high efficacy through cell wall/ insect cuticle hydrolysis [16,25,51,71,73,136]. Moreover, the dual antimicrobial and insecticidal efficacy of hydrolytic enzymes produced by some *Bacillus* sp. demonstrates the prospect for the simultaneous biocontrol of phytopathogenic infections and insect pests in both nursery and tree plantations. For instance, in our previous studies we demonstrated the potential of *B. velezensis* CE100 [51,53,54,55,72,73] and *B. licheniformis* PR2 [60,71,105] as effective biocontrol agents against fungal diseases and insect pests.

### 4.2. The Biocontrol Prospect of Hydrolytic Enzymes from Bacillus sp. against Viral Diseases and Virus-Transmitting Vectors

Unlike the biocontrol of fungal/oomycete and bacterial diseases, there is relatively little research progress on the biocontrol prospect of viral infections in plants. Nonetheless, some studies have indicated the potential of biocontrol of *Bacillus* sp. against viral diseases, especially through induced systemic resistance (ISR) or systemic acquired resistance (SAR) against plant viral infections and virus vectors [170,171,172]. For instance, *B. amyloliquefaciens* strain VB7 was reported to reduce the incidence (leaf lesions) of tobacco streak virus (TSV) from 25.28 lesions cm^−2^ area/leaf in the control to 2.22 lesions cm^−2^ area/leaf and lowered the field incidence rate by 52% while simultaneously increasing cotton yield by approximately 53% [173]. The elicitor compounds for ISR secreted by the *Bacillus* sp. against viral infections have not been precisely established but there is evidence of increased pathogen-related proteins (PRPs) such as chitinases and glucanases mainly through the salicylic-dependent signaling pathway. This may involve, among other processes, the manipulation of the cell wall composition and biosynthesis of phytoalexins to facilitate disease resistance in plants against a broad spectrum of phytopathogenic groups, including plant viruses [174,175]. The role of beneficial microbes such as *Bacillus* sp. in inducing defense responses in plants often proceed through microbe-associated molecular patterns (MAMPs) including lipopolysaccharides, where, unlike the pathogen-associated molecular patterns (PAMPs), the MAMPs stimulate host immune mechanisms without causing cellular damage [175]. Abdelkhalek et al. [176] reported that the inoculation of *B. licheniformis* strain POT1 in potato plants stimulated the production of polyphenols, and the expression of the enzyme (*F3H*) genes responsible for the biosynthesis of flavonoids in plants as well as the anthocyanin 2 transcription factor such as anthocyanin, which are considered major antiviral defense factors. In their study, pyrrolo [1,2-a]pyrazine-1,4-dione was suggested as a major elicitor for inducing SAR against alfalfa mosaic virus (AMV) in potato. Guo et al. [177] also demonstrated that *B. amyloliquefaciens* strain Ba13 induces plant systemic resistance against tomato yellow leaf curl virus (TYLCV) through the activity of PRPs, as evidenced by the elevated expression of *PR1*, *2* and *3* genes, the increased the activity of plant defense enzymes such as phenylalanine ammonia lyase (PAL), polyphenol oxidase (PPO), peroxidase (POD), β-1,3 glucanase, and chitinase, which are associated with both pathogenesis and herbivorous resistance in plants. Wang et al. [173] demonstrated the role of *Bacillus* sp. in inducing defense response against tobacco mosaic virus (TMV) through the jasmonate-mediated signaling pathway (*Coi1* gene) and *nonexpressor of pathogenesis-related genes1* (*NPR1*), and *pathogenesis-related -1a and -1b* genes (*PR-1a* and -*1b*). The insecticidal role of hydrolytic enzymes secreted by *Bacillus* sp. against plant virus vectors, mainly hemipteran insect pests such as aphids and whiteflies through the degradation of exoskeleton cuticles and peritrophic membrane has been described [108,110,120,146,147]. A detailed review of the role of *Bacillus* sp. in eliciting plant defense responses has been reported [178,179]. However, future research should also focus on the direct role of hydrolytic enzymes (especially glucanases and chitinases) on the potential to induce host resistance against viral infections, since they have been demonstrated to play a vital role in induced and acquired defense mechanisms.

## 5. Conclusions

Hydrolytic enzymes are produced by *Bacillus* sp. in high concentrations in the bacterial culture and their dual antimicrobial and insecticidal efficacy has been demonstrated in purified fractions, crude fractions, and in the bacterial culture, which makes their application less complex and economically more viable compared to antimicrobial lipopeptides that require complex purification process to attain effective concentrations. The major antifungal/anti-oomycete mechanism by hydrolytic enzymes involves the lysis of the phytopathogenic cell walls in the spore or mycelial cells, which leads to the inhibition of spore germination, suppression of mycelial growth and germ tube elongation and prevention of pathogenic attachment and appressoria formation to control infections. Similarly, the insecticidal activity of the lytic enzymes from *Bacillus* sp. is mainly based on the hydrolysis of the lipid (wax coating), chitin fibrils and structural proteins (mainly glycoproteins), leading to the degradation of the cuticle layer and cuticular appendages such as setae. This causes desiccation and exposes internal organs to environmental aggressions (pressure, infection, and toxins), which causes insect mortality or reduces the ecological fitness of surviving insects, leading to reduced herbivore activity of insect pest/plant damage. The dual insecticidal/fungicidal activity of hydrolytic enzymes from *Bacillus* sp., through cuticle/cell wall degradation, could be further studied to develop biocontrol products for the simultaneous control of insect pests and fungal diseases in forest and fruit tree production. The hydrolytic enzymes from *Bacillus* have also demonstrated an indirect effect against plant viral infections by controlling virus vectors such as hemipteran insect pests and by inducing plant defense responses that reduce the viral load, and ultimately suppress the incidence and severity of viral symptoms. The production of hydrolytic enzymes from *Bacillus* sp. using locally available (cost-effective) sources of energy (fermentation media), such as crab shells and other chitin-containing substances as carbon sources, and their mass cultivation using on-farm fermentation systems is a key component to their successful application and their adoption as an eco-friendly strategy of pest and disease management at various levels of production. Thus, future studies should aim at the optimization of the fermentation media for *Bacillus* sp. (using cost-effective energy sources) to produce high and stable concentrations of hydrolytic enzymes and investigating the prospect for the simultaneous biocontrol of phytopathogens and insect pests (including plant parasitic nematodes), which have not been fully explored in forestry and fruit tree production systems. Besides the reports about novel isolations and their characterization, future research should also investigate the potential synergism of co-inoculating different species or strains with varying spectra of hydrolytic enzyme activity that could improve the utilization of *Bacillus* sp. as biological control alternatives to chemical pesticides, especially for the simultaneous control of fungal diseases and insect pests in nursery and field plantations.

## Figures and Tables

**Table 1:** The role hydrolytic enzymes from *Bacillus* sp. in the biocontrol of phytopathogens/insect pests of potential economic significance.

*Bacillus* Species	Enzyme (s)	Pathogen/Insect Pest (Disease/Damage)	Host Plant	Reference
*B. velezensis* CE100	Chitinase, protease, β-1,3-glucanase	*Colletotrichum gloeosporioides* (anthracnose)	*Juglans regia* L.	[51]
	Chitinase, β-1,3-glucanase	*Macrophomina phaseolina*, *Fusarium oxysporum* f. sp. *fragariae* (charcoal rot, wilt)	*Fragaria × ananassa*	[55]
	Chitinase, protease, β-1,3-glucanase	*Pestalotiopsis maculans* (*Pestalotiopsis* blight)	*Quercus acutissima*	[53]
	Protease, β-1,3-glucanase	*Phytophthora* species (*Phytophthora* wilt)	*Chamaecyparis obtusa*	[54]
	Chitinase, protease	*Dasineura jujubifolia* larvae (leaf roll damage)	*Ziziphus jujuba*	[73]
	Chitinase, protease	*Reticulitermes speratus kyushuensis* (wood tunneling)	*Pinus densiflora*	[72]
*B. licheniformis* MH48	Chitinase, β-1,3-glucanase	*F. oxysporum* (root rot)	*Pinus thunbergii*	[106]
	Chitinase, β-1,3-glucanase	*B. cinerea, Glomerella cingulata, P. diospyri, and P. karstenii* (foliar diseases)	*Camellia oleifera*	[107]
*B. licheniformis* PR2	Chitinase	*B. cinerea*, *C.gloeosporioides*, *Phytophthora nicotianae* (fruit rot)	*Zizyphus jujua*	[105]
	Chitinase, protease	*R. speratus kyushuensis* (wood tunneling)	*P. densiflora*	[60]
	Chitinase, proteases	*H. cunea* larvae (defoliation)	*Populus × canadensis*	[71]
*Bacillus subtilis*	Glucanase, proteases	*Rosellinia necatri*, *F. oxysporum* f.sp. *radicis-lycopersici* (root rot)	*Persea americana*, *Solanum lycopersicum*	[141]
*B. cereus* 108	Chitinase	*F. solani* (wilt disease)	*S. lycopersicum*	[90]
*B. pumilus* SG2	Chitinases (ChiS, ChiL)	*R. solani*, *Verticillium* sp. and *Stemphyllium botryosum* (blight and wilt diseases)	Fruit trees	[91]
*B. licheniformis* MY75	Chitinase	*Gibberella saubinetii* (perfect stage of *F. graminearum*), *A. niger* (head blight, black mold)	Cereals, fruits	[92]
*B. amyloliquefaciens* AG1	Protease-like	*Aspergillus* sp., *B. cinerea*, *F. oxysporum, V. dahlia* (grape decay, wilt)	*Vitis vinifera*	[162]
*B.licheniformis* BS-3	Protease	*Aspergillus niger*, *M. oryzae*, *Rhizoctonia solani*	NS	[125]
*Bacillus cereus* 28-9	Chitinase	*B. elliptica*	NS	[89]
*B. thuringiensis* NM101-19 *B. licheniformis* NM120-17	Chitinase	*Rhizoctonia* sp., *F. oxysporum*, *Penicillum chrysogenum* (wilt, mold, mycotoxins)	*Glycine max*	[70]
*B. subtilis* TV-125	Chitinase	*F. culmorum*	NS	[96]
*B. amyloliquefaciens* V656	Chitinase	*F. oxysporum*	NS	[56]
*B. subtilis* NPU 001	Chitinase	*F. oxysporum*	NS	[58]
*B. cereus* YQ308	chitinase	*F. oxysporum* and *P. ultimum*	NS	[57]
*B. thuringiensis* var *israelensis*	Chitinase	*S. rolfsii*, *Rhizopus* sp., *Fusarium* sp., *Aspergillus* sp. (wilt)	*G. max*	[88]
*B. cereus* NK91	Chitinase	*F. oxysporum*, *R. solani*, and *C. gloeosporioides*	NS	[94]
*B. licheniformis* J24	Chitinase	*F. pseudograminearum* (Fusarium rot)	*Zea mays* seeds	[95]
*B. subtilis* TV-125	Chitinase	*F. culmorum* (root rot)	Vegetables	[96]
*B. subtilis*	Chitinase	*A. niger*, *A. flavus*, and *P. chrysogenum*	NS	[97]
*Bacillus* sp. 739	Chitinase	*Fusarium* sp. and *H. sativum*	NS	[98]
*B. velezensis* RB.DS29	Protease, β-glucanase, chitinase	*Phytophthora* sp. (root rot disease)	*Piper nigrum*	[163]
*B.cereus* QQ308	Chitinase, chitosanase, protease	*F. oxysporum*, *F. solani*, and *P. ultimum* (root, head/soft rot disease)	*B. rapa*	[59]
*B. subtilis* SL-13	Chitinase	*R. solani* (foot rot)	*S. lycopersicum*	[93]
*B. pumilus* RST25	Chitinase	*F. solani* and *A. niger* (seed rot)	*Triticum aestivum*	[99]
*B. chitinolyticus* (SGE2, 4, SSL3), *B. ehimensis* MG1	Chitinase	*A. nidulans*, *B. cinerea*, *F. culmorum*, *S. sclerotiorum*, *Guignardia bidwellii*	NS	[100]
*B. licheniformis* TCCC10016	Chitinase	*F. oxysporum*	NS	[101]
*B. pumilus* CCIBP-C5	Chitinase	*Pseudocercospora fijiensis* (black sigatoka)	*Musa* sp.	[102]
*B. cereus sensu lato* B25	Exochitinase A, endochitinase B	*F. verticillioides* (rot and wilt diseases)	*Z. mays*	[103]
*B. licheniformis* ATCC 14580	Chitinase	*Phoma medicaginis* (damping-off)	*Medicago truncatula*	[104]
*Bacillus* sp.	amylase, protease, pectinase, cellulase	*F. equiseti* (*Fusarium* wilt)	*Vicia faba*	[164]
*Bacillus subtilis*	Glucanase, protease	*F. oxysporum* f.sp. *radicis-lycopersici* (root, crown rot)	*S. lycopersicum*	[141]
*B. cereus* 1.21	Chitinase	*Bemisia tabaci* (sap sacking/leaf curling)	*C. annuum*	[108]
*B. licheniformis* USMW10IK	Chitinase	*Globitermes sulphureus* (wood tunneling)	Wood, trees	[109]
*B. cereus* C-13	Chitinase	*H. theivora* (sap-sacking)	*C. sinensis*	[110]
*B. subtilis*	Chitinases (CS1, CS2)	*S. litura* larvae (defoliation)	*Nicotiana tabacum*	[113,114]
*B. thuringiensis* subsp. *israelensis* IPS78	Exochitinase	*C. nubeculosus* larvae	NS	[115]
*B. thuringiensis* subsp. *aizawai* HD133, HD-1	Exochitinase	*S. littoralis*, *S. litura* (leaf damage)	*S. lycopersicum*	[115,117]
*Bacillus* sp. RH219	Proteases Apr219, Npr219	*Panagrellus redivivus* nematodes	NS	[132]
*B. nematocida* B16	Protease Bae16	*P. redivivus*, *Bursaphelenchus xylophilus* (pine wilt)	*Pinus* sp.	[133]
*Bacillus* sp. B16	Serine protease	*P. redivivus*	NS	[130]
*B. cereus* NJSZ-13	Alkaline protease	*B. xylophilus* (wilt disease)	*P. elliottii*	[135]
*B. licheniformis* W10	Serine protease	*B. cinerea*	NS	[122]
*B. licheniformis* TG116	Serine protease	*P. capsica*, *R. solani*, *F. graminearum*, *F. oxysporum*, *B. cinerea*	NS	[123]
*B. subtilis* 21	Protease	*F. verticillioides, R. solani* (wilt, black root rot)	*F. ananassa*	[124]
*B. velezensis* ZJ20	β-1, 3-1, 4-glucanases	*Cryphonectria parasitica*, *Helicobasidium purpureum*, *Cylindrocladium quinqueseptatum*	NS	[137]
*B. subtilis* CW14	β-glucanase	*A. ochraceus* (mold, ochratoxins)	*G. max*	[138]
*B. amyloliquefaciens* MET0908	β-1,3-glucanase	*C. lagenarium* (anthracnose)	*Citrullus lanatus*	[139]
*B. subtilis* NSRS 89-24	β-1,3-glucanase	*Pyricularia grisea*, *R. solani* (rice blast, sheath blight)	*Oryza sativa*	[140]
*Bacillus* sp. strain 739	Lipase, chitinase, protease, β-1,3-glucanase	*Bipolaris sorokiniana* (root rot)	Cereal	[143]
*B. subtilis* AI01, AI03	Lipase, protease	*F. solani* (*Fusarium* wilt)	*Solanum melongena*	[145]
*B. subtilis* Ehrenberg	Lipases	*Planococcus citri* (sap sucking)	Citrus	[146]
*B. subtilis*	Lipase	*Maconellicoccus hirsutus* (sap-sucking)	*Gossypium* sp., *V. vinifera*, *Z. jujuba*.	[147]
*Bacillus* sp. KD7	Amylase	*A. flavus* (mold and mycotoxin)	Cereals	[150]
*B. subtilis* GM2, GM5	Amylase	*Fusarium* sp. (wilt disease)	*Triticum* sp. seedling	[151]
*B. velezensis* HY-3479	Amylase	*C. acutatum*, *Cylindrocarpon destructans*, *R. solani*, *S. sclerotiorum*	NS	[152]
*B. licheniformis*	Amylase	*V. dahlia*, *F. oxysporum*, *Phytophthora* sp., *C. acutatum*, *B. cinerea, Aspergillus* sp.	NS	[153]
*B. subtilis* B71	Cellulase	*P. spinosium*	NS	[155]
*B. subtilis* EG21	Cellulase, pectinase, chitinase	*P. infestans* (blight disease)	*Solanum tuberosum*	[156]
*B. amyloliquefaciens* UQ154, *B. velezensis* UQ156	Cellulase, protease	*Phytophthora* sp. (*Phytophthora* blight)	*C. annuum*	[157]
*B. velezensis* 6-5	Cellulase	*P. infestans* (blight disease)	*S. tuberosum*	[158]
*B. pumilus*	Cellulase, protease, mannanase	*P. ultimum, Aphanomyces cochleoides*	NS	[159]
*B. licheniformis* BL06	Cellulase	*P. capsica* (*Phytophthora* blight)	*C. annuum*	[160]
*B. velezensis* SN337	Cellulase	*Phytophthora sojae* (root rot)	*G. max*	[161]

NS indicates that the in vivo study was not conducted.

## Data Availability

All the reviewed articles can be accessed by searching the references listed below.
